# Research Involvement and Engagement: reflections so far and future directions

**DOI:** 10.1186/s40900-017-0074-y

**Published:** 2017-11-08

**Authors:** Richard Stephens, Sophie Staniszewska

**Affiliations:** 1 Involved and engaged patient and carer, Stevenage, UK; 2 Warwick Research in Nursing, University of Warwick Medical School, Coventry, UK

## Abstract

**Plain English summary:**

Two years ago we launched *Research Involvement and Engagement* (RIE) as an interdisciplinary co-produced journal, focusing on patient and wider involvement and engagement in all stages of health and social care research. In this Editorial we reflect on progress and consider future directions. Now indexed in PubMed Central, RIE’s prime objective is to publish papers that report public involvement in enough depth to generate a sound and robust evidence base, from which others can draw to develop best practice. Our open access publishing enables anyone who wants to read a paper to access it free of charge, a powerful way of making research more open and more democratic, with RIE a key part of this slow but necessary revolution. While we have made progress, there is still a long way to go to embed involvement and engagement as normal within research practice. Publishers and funders have a vital role to play in changing research so the co-production of knowledge becomes the norm. In this Editorial we highlight key areas that we need to develop to strengthen involvement and engagement. We draw strength from knowing we are not alone in this journey. Our Editorial Board, our authors, our reviewers, and you dear readers, are all companions on this journey, making a wide range of contributions that help us move forward, slowly but surely.

**Abstract:**

Two years ago we launched *Research Involvement and Engagement* (RIE) as an interdisciplinary co-produced journal, focusing on patient and wider involvement and engagement in all stages of health and social care research. In this Editorial we reflect on progress and consider future directions. Now indexed in PubMed Central, RIE’s prime objective is to publish papers that report public involvement in enough depth to generate a sound and robust evidence base, from which others can draw to develop best practice. Our open access publishing enables anyone who wants to read a paper to access it free of charge, a powerful way of making research more open and more democratic, with RIE a key part of this slow but necessary revolution. While we have made progress, there is still a long way to go to embed involvement and engagement as normal within research practice. Publishers and funders have a vital role to play in changing research so the co-production of knowledge becomes the norm. In this Editorial we highlight key areas that we need to develop to strengthen involvement and engagement. We draw strength from knowing we are not alone in this journey. Our Editorial Board, our authors, our reviewers, and you dear readers, are all companions on this journey, making a wide range of contributions that help us move forward, slowly but surely.

## Introduction

Two years ago we launched *Research Involvement and Engagement* (RIE) as an interdisciplinary co-produced journal, focusing on patient and wider involvement and engagement in all stages of health and social care research. The launch of RIE both reflected and stimulated the increasing international interest in patient and public involvement in research. RIE was and still is the only co-produced academic journal on this theme, and the only international journal co-produced by all key stakeholders, including patients, academics, policy makers and service users.

We are very pleased to announce that RIE is now indexed in PubMed Central. With this significant milestone, which means we will reach a much wider audience and help us develop our international profile, we thought it timely to reflect on where we are and where we are going.

## Where are we?

RIE’s prime objective is to publish papers that report public involvement in enough depth to generate a sound and robust evidence base, from which others can draw to develop best practice. However we acknowledge that the interest in involvement and engagement also reflects broader changes in societies towards active citizenship, the democratisation of knowledge and research, and a more consumerist view of research outputs from the public.

So RIE welcomes a wide range of papers and articles from anyone involved or engaged with research, looking into supporting, encouraging or delivering the patient/public voice in research processes or structures. Our motivation is based on our evidence-informed perspective that research really is better when it involves everyone who needs or wants a voice, helping ensure the focus of a study is relevant, acceptable and of the highest quality, for all our benefit.

Every paper that we consider for publication is reviewed by both patients and researchers, as we recognize that both bring valuable but often different perspectives. Our open access publishing enables anyone who wants to read a paper to access it free of charge, and our open peer review policy means that readers can also see the discussions and debates that have occurred between authors and reviewers, which are often a rich source of nuanced opinions, sometimes more trenchantly expressed than in the published paper.

Our innovative requirement for authors to include a Plain English summary at the point of initial submission is intended to ensure that the paper’s contents are accessible to “the reasonably well-educated and/or well-informed patient and those who are on this journey.” It has the added benefit of allowing simpler online translations for patients in other countries. The “reasonably well-educated patient” is one of our target audiences, in all nations not only the English-speaking ones. We are pleased that so many of the graduates of the European Patients Academy (EUPATI) have accepted our invitation to become patient reviewers, and that the papers in our journal are helping to inform and support patient involvement and engagement in research in diseases, healthcare systems and cultures where it is still a very novel experience for all parties.

We are different and we celebrate that difference, hoping to inspire others to consider the potential for involvement and engagement in other fields.

Two years on from the launch of RIE is a good point to review progress, address challenges and consider the future. We have published 38 research papers, five methodologies, six reviews, 10 commentaries, three protocols and four other contributions (data as of 29 September 2017). We are undertaking a survey of patient reviewers jointly with *The BMJ* to understand the peer review experience from the perspective of patients, so we can improve this experience. So, lots of activity, lots of papers and a good time to think about the challenges and opportunities and our ambition for the journal.

## Where are we going?

While involvement and engagement is supported by many funders, researchers, policy makers, patients and members of the public, we still have some way to go to embed it as a customary feature of the research process. Involvement can still exist as an add-on, and perhaps this a particular risk where funders insist on having patient or public involvement or engagement as a requirement for funding the study, but without necessarily encouraging researchers to view it as an evidence-informed activity that often requires an evaluation of mechanism and outcomes. Such evaluations help build evidence-based practice, enabling those undertaking the research to focus their efforts on effective forms of involvement. We encourage researchers to submit papers to RIE to share the methodologies of their evaluations and/or the outcomes of their impact assessments. We also encourage relevant contributions from funders and policy-makers.

But if in its worst form patient involvement may still be only a tick box exercise, in its best form it can be transformational, with teams of researchers and patients co-producing new knowledge that has a positive impact on peoples’ lives. Somewhere between the two extremes the submissions sent to RIE contain many examples of involvement and engagement at specific but isolated points in a piece of research. These are typically focus groups decoding priorities from a list, or “expert” patients reviewing materials or similar activities. They are the staple diet of engagement and involvement perhaps, but they can often represent opportunities missed as much as those taken.

We still have a long way to go to achieve the paradigm change required for involvement and engagement to be ‘business as usual,’ but each paper in RIE represents a small step forwards and adds to an evidence base that researchers, patients, practitioners and public members can use for effective practice. Over time we may find that the content of research changes, reflecting the issues of importance to patients and the public [1].

The papers we have published in RIE usually deal with the stuff of knowledge, the content or information about an area of research. Less often is emphasis placed on the need to involve patients and the public in developing standard methods and methodologies, the bones of research. This would ensure that we embed involvement and engagement within the way we work, so that patients and researchers working together becomes business as usual. One very promising area is the field of patient reported outcomes, where some researchers acknowledge the need to collaborate with patients in creating new methods, concepts and methodologies that support active forms of involvement [2].

Funders need to support such initiatives by recognising their critical role in achieving a paradigm shift in the nature of research, creating new ways of working that share the power of decision-making. While this may be seen as radical, it would be an important step towards co-production of knowledge and evidence. In this context we look forward at RIE to receiving submissions from researchers working in industry, who are increasingly exploring crowd-sourcing and the use of social media and modern technology to inform their work. The boundaries between medical research and market research may be blurring, and as noted above, in an increasingly consumerist world, patients regard themselves as research participants not subjects, and they are not only service-users but product-users too.

While RIE is an academic journal publishing high quality research, we recognise that involvement and engagement is a young and evolving area of endeavour. We expect and encourage a wide range of papers, some reporting substantive studies while others might report on a 1 day meeting to agree priorities in an area of research. Both of these endeavours are important to us, as they each contribute in their own way to the evidence-base jigsaw, but we recognise that some in traditional academia view the latter as being of poorer quality.

We understand that view but we do not share it. On the contrary we recognise that the evidence base is evolving and our view is that RIE is itself part of that evolution. We do not simply record or report, we seek to inspire and support. We have nurtured these “academically less strong” papers, helping authors draw on peer review comments to enhance the quality of their paper. For us these papers often report knowledge spaces, conversations and contributions that represent the building blocks of involvement and engagement. In some cases the papers have been in review, and in re-review, and even in re-re-review, for many months. In some cases papers are too poor to enter the review process, but we encourage authors to develop their thinking and submit another day, and if we can, we offer guidance on how they may do this.

We thank all of our authors and especially our reviewers who have contributed their time and energy to this work. Your commitment is one of the facets that makes RIE a unique journal. We will continue RIE’s editorial policy of trying to find something of interest and of value in every paper we receive, if it is possible to do so. That certainly makes us different from other academic journals, although it can sometimes mean that papers spend a long time in the review-revision process, and for some published papers, the comments and discussions between reviewers and authors, available via our open-access review policy, are as enlightening as the paper itself.

In the long term however, our community needs to elevate the number and quality of involvement and engagement papers, and funders internationally have a key role in recognising the legitimacy of funding involvement and engagement studies in their own right. We need substantive evidence-generating studies that create new knowledge about involvement and engagement, alongside the more routinely supported involvement activity, the ‘doing’ activities, which although valuable, do not always generate research-based knowledge that push knowledge forward. “Adding a research element to the ‘doing’ parts of studies could be an important way forward. Research into research” is not always a priority for funders, but to support such work in involvement and engagement is in their own interests.

A final challenge is enhancing the quality of reporting. We need researchers to recognise that it is important to report their PPI activity, even if feels like an additional element to their main focus. In this way, we can ensure we avoid research waste, when key information may be collected in a study but then omitted in a final paper.

Our recent publication of GRIPP2, EQUATOR guidance for reporting involvement, represents a step in the right direction [3, 4]. We need to change the inconsistent nature of involvement and engagement reporting towards a more consistently reported set of information. We would encourage our authors to consider using GRIPP2, either the short form or the long form, with a reminder that items are not mandatory, but that they aim to encourage careful reflection and to support the researcher and patient in making the most of reporting their study.

Key organisations have come together recently to create the beginnings of a new international network including the University of Warwick, Comet, Cochrane and the National Institute for Health Research. We hope that this network provides a new priority to elevate involvement and engagement internationally, gaining strength from joining forces, creating new opportunities for linking together, identifying common challenges and creating solutions, in an attempt to create the paradigm change required in academic research. We look forward to receiving their submissions for publication in RIE.

## Conclusions

The journal of *Research Involvement and Engagement* will continue to address the significant challenges and the enormous opportunities that we have described. We want to grow, to attract international papers, and to enhance the quality of what we publish. We want to support researchers, patients and the public. We want to celebrate the great potential of co-creating knowledge and evidence. We want involvement and engagement to be seen as integral to definitions of research quality. We thank all our authors, reviewers and the publishers who have collectively believed in this movement. We hope that you, dear reader, will join us.
